# Vegetarian and Vegan Weaning of the Infant: How Common and How Evidence-Based? A Population-Based Survey and Narrative Review

**DOI:** 10.3390/ijerph17134835

**Published:** 2020-07-05

**Authors:** Maria Elisabetta Baldassarre, Raffaella Panza, Ilaria Farella, Domenico Posa, Manuela Capozza, Antonio Di Mauro, Nicola Laforgia

**Affiliations:** 1 Department of Biomedical Science and Human Oncology, Neonatology and Neonatal Intensive Care Unit, “Aldo Moro” University of Bari, 70100 Bari, Italy; domenico.posa@gmail.com (D.P.); manuela.capozza@uniba.it (M.C.); antonio.dimauro@uniba.it (A.D.M.); nicola.laforgia@uniba.it (N.L.); 2 Department of Pediatrics, Queen Fabiola Children’s University Hospital, 1020 Bruxelles, Belgium; ilafarella@yahoo.com

**Keywords:** weaning, infant, vegetarian diet, vegan diet, complementary feeding

## Abstract

Background: Vegetarian and vegan weaning have increasing popularity among parents and families. However, if not correctly managed, they may lead to wrong feeding regimens, causing severe nutritional deficiencies requiring specific nutritional support or even the need for hospitalization. Aim: To assess the prevalence of vegetarian and vegan weaning among Italian families and to provide an up-to-date narrative review of supporting evidence. Materials and methods: We investigated 360 Italian families using a 40-item questionnaire. The narrative review was conducted searching scientific databases for articles reporting on vegetarian and vegan weaning. Results: 8.6% of mothers follow an alternative feeding regimen and 9.2% of infants were weaned according to a vegetarian or vegan diet. The breastfeeding duration was longer in vegetarian/vegan infants (15.8 vs. 9.7 months; *p* < 0.0001). Almost half of parents (45.2%) claim that their pediatrician was unable to provide sufficient information and adequate indications regarding unconventional weaning and 77.4% of parents reported the pediatrician’s resistance towards alternative weaning methods. Nine studies were suitable for the review process. The vast majority of authors agree on the fact that vegetarian and vegan weaning may cause severe nutritional deficiencies, whose detrimental effects are particularly significant in the early stages of life. Discussion and conclusion: Our results show that alternative weaning methods are followed by a significant number of families; in half of the cases, the family pediatrician was not perceived as an appropriate guide in this delicate process. To date, consistent findings to support both the safety and feasibility of alternative weaning methods are still lacking. Since the risk of nutritional deficiencies in the early stages of life is high, pediatricians have a pivotal role in guiding parents and advising them on the most appropriate and complete diet regimen during childhood. Efforts should be made to enhance nutritional understanding among pediatricians as an unsupervised vegetarian or vegan diet can cause severe nutritional deficiencies with possible detrimental long-term effects.

## 1. Background

Over the last decades, vegetarian and vegan diets have become more popular worldwide, with a reported increase in prevalence of 350% [1]. Hence, it is no longer uncommon for a pediatrician to be asked by parents for vegetarian- or vegan-based weaning. As a consequence, it is crucial for pediatricians to gain a deep and extensive knowledge of both vegetarian and vegan diets to avoid any associated risks, since the limited variety of foods of both the vegetarian and vegan diet makes it difficult to meet the correct intake of all nutrients needed by the infant, with significant chances of nutritional deficiencies. Families adopt a vegetarian/vegan diet for many motivations: according to a recent Italian review, 47.6% of families believe that vegetable proteins are healthier than animal protein, while 31.7% are vegetarian for environmental reasons. The remaining cases can be ascribed to religious, philosophical and economic reasons [2].

As for adults, 5% of North Americans are vegetarian and 3.7% vegan; the prevalence of vegetarianism is higher in Australia (11.2%) and reaches a peak in India where 30% of the population are vegetarian, mainly for religious reasons [1]. In Europe, the prevalence of vegetarianism is also surging, with differences among nations, i.e., 2% in France, 9% in Germany, 10% in Italy and 12% in the UK [1]. While epidemiological data on eating habits in adulthood are largely available, they are still scarce for the pediatric population. Epidemiological data in childhood are available only from the North American organization, the Vegetarian Resource Group, that reported 1% prevalence of veganism in children [3].

There are different types of vegetarian diets. By definition, a vegetarian diet excludes consumption of all types of meat, meat products, fish, and clams. Dairy products, eggs, and honey are permitted. Conversely, semi-vegetarianism allows all kinds of foods (meat included) to be consumed, but with some limits of both quantity and frequency. 

There are two main types of vegetarian diet:

- Lacto-ovo-vegetarianism, which encompasses dairy products, eggs, and honey. Subcategories are lacto-vegetarianism that prohibits eggs, and ovo-vegetarianism that prohibits dairy products;
- Veganism, that prohibits dairy products, meat, eggs, and honey. All vegetables, seaweed, mushroom and bacteria (probiotics) are allowed.

Less commonly, people follow other plant-based diets, such as:

- Raw food diet, which consists of vegetables, legumes and pulses, fruits, cereals, seeds, milk and eggs, all of which are mainly consumed raw;
- Fruit diet, which consists mainly of fresh and dried fruits, but allows also seeds and some vegetables;
- Macrobiotic diet, which is based on cereals, vegetables, legumes and pulses, seaweed, and soy products; fish may also be consumed [4].

Since vegetarian and vegan diets allow a limited variety of foods, neonates whose mothers are vegetarian/vegan and infants following alternative weaning methods may be exposed to clinical or sub-clinical nutritional deficiencies.

### Aim

The aim of the present study is to assess the prevalence of vegetarian and vegan diet among Italian families, and how often they choose an alternative weaning for their children, and also to provide an up-to-date narrative review of supporting evidence for vegetarian and vegan weaning.

## 2. Materials and Methods

### 2.1. Survey

A questionnaire made of 40 questions was submitted to Italian families by the No-Profit Association “La Medicina in uno Scatto” in collaboration with Maria Elisabetta Baldassare, Associate Professor in Pediatrics, from January 2019 to December 2019. In a first step, 1000 primary care pediatricians were randomly selected from the mailing list of the Italian Federation of Pediatricians (Federazione Italiana Medici Pediatri, FIMP). An invitation email was then sent out to the selected pediatricians to participate anonymously in a larger study assessing both weaning methods in ex-preterm infants [5] and the prevalence of alternative weaning regimens among their patients. For the latter aim, pediatricians were asked to forward the specific questionnaire to randomly selected voluntary families.

The topics covered by the 40 items were:

(1) The prevalence of alternative weaning;
(2) The correlation between parental food regimens and the type of weaning;
(3) The food regimen chosen by children at the time of autonomous food selection;
(4) Breastfeeding duration according to the type of weaning;
(5) The role of family pediatricians in the management of weaning;
(6) The use of food supplements.

The questionnaire was formulated according to the guidelines published by many international pediatric societies, i.e., the American Academy of Pediatrics (AAP), the Academy of Nutrition and Dietetics (ADA), the European Society for Pediatric Gastroenterology Hepatology and Nutrition (ESPGHAN) and the Italian Pediatric Society (SIP). 

Data were uploaded as spreadsheet using Microsoft Excel, version 16.30, and analyzed by Stata MP11 software (StataCorp LLC, 4905 Lakeway Drive, College Station, TX, USA). Categorical variables were reported as proportions. Quantitative variables were described as means ± standard deviations (SD) and median. When appropriate, the significance of the differences was calculated using the chi-square test. A p-value of less than 0.05 was considered statistically significant.

The participation was voluntary and written consent was not needed. Ethical approval was not sought since all data were anonymous.

### 2.2. Review

An exhaustive search for eligible studies was performed in PubMed, Embase, Medline, Cochrane library and Web of Science databases. The following Medical Subject Headings (Mesh) were used: “Weaning” [Mesh], “Infant, newborn” [Mesh], “Diet, vegetarian” [Mesh], “Diet, vegan” [Mesh], “Complementary feeding [Mesh]”. Proper Boolean operators “AND” and “OR” were also included to be as comprehensive as possible. Additional studies were sought using references in articles retrieved from searches. Studies were eligible if they were primarily focused on the impact of vegetarian and vegan diets throughout the first year of life. We excluded papers on vegetarian and vegan feeding regimen exclusively during pregnancy or later in childhood and papers on raw food diet, fruit diet and macrobiotic diet, because they are very rare in our study population. Search limits were set for studies published between 1st May 2010 and 30th April 2020 in the English language.

Once the criteria have been applied, four of the resulting papers were eligible, but one was excluded because it was on complementary feeding in general [6] and another one on vitamin B12 deficiency due to reduced maternal Vitamin B12 status not related to diet [7]. Seven more papers have been added from references of articles retrieved from initial search, so that nine were finally selected for this review. 

**Figure 1:**
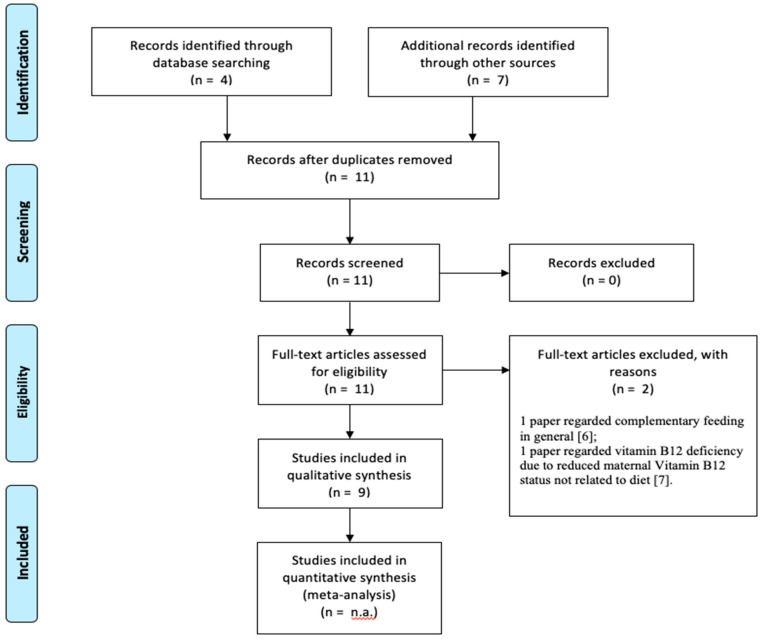
Study selection according to PRISMA guidelines.

The selection process was conducted according to PRISMA guidelines (Figure 1).

## 3. Results

### 3.1. Survey

The survey has been completed by 360 Italian families. Demographics are shown in Table 1.

The results of our survey are reported in Table 2. 

An omnivore weaning regimen was adopted in 90.8% infants, while alternative weaning was followed in 9.2% infants. Vegetarian/vegan mothers were 8.6%. Furthermore, unconventional weaning was associated to a longer breastfeeding period compared to omnivore weaning (15.8 vs. 9.7 months; *p* < 0.0001).

Weaning and parental diet are reported in Table 3.

We also evaluated how the family pediatrician has been involved in the weaning process. Among parents who weaned their child according to a vegetarian regimen, 77.4% informed and consulted the family pediatrician, while 22.6% followed a vegetarian weaning without any consult. The pediatrician was considered sufficiently knowledgeable about unconventional weaning by 54.8% of parents, whereas 45.2% of parents considered their pediatrician unable to give adequate information. When parents consulted family pediatrician, 77.4% of them encountered pediatricians’ opposition. However, 96.8% continued to attend scheduled pediatric visits regularly. Multivitamin supplementations according to weaning type were administered to a similar extent in the two groups (85.1% in omnivore-weaned and 87.1% in vegetarian-weaned infants; *p* = 0.76). Only 37.7% of vegetarian-weaned infants were given vitamin B12 supplementation.

### 3.2. Narrative Review 

The present review included nine studies: five recommendations/position papers [4,8,9,10,11], one case report [12], one brief report [13], one review [14], and one editorial [1]. Neither randomized controlled trials nor observational studies were found (Table 4).

As in Table 5, data on vegetarian or vegan diets refer to different age groups: case reports are mainly focused on the first year of life [12,13], whereas recommendations and position papers [4,8,14] address feeding regimens throughout the lifespan. ESPGHAN position paper is focused on complementary feeding with a brief comment on alternative weaning regimens [10]. 

Despite different findings, the vast majority of authors agree on the fact that vegetarian and vegan weaning may cause severe nutritional deficiencies [1,8,10,12,13], whose detrimental effects are particularly severe if the deficiency starts early in life, as in infants breastfed by vegetarian/vegan mothers or bottle-fed with nondairy drinks [13].

Psychomotor regression with bone fractures due to nutritional rickets (vitamin D deficiency) [12], hospitalizations for severe vitamin B12 deficiency [15], deep hypocalcemia with seizures, severe anemia, respiratory distress with metabolic alkalosis, growth retardation, and death [13] have been reported in cases of alternative feeding regimens during the first two years of life.

Recommendations and position papers [4,8,9,14] report the main concerns of vegetarian and vegan diets during the early phases of life, as summarized below.

• Protein intake

Protein needs during the first year of life range between 1.2 and 2.2 g/kg/day, being higher from 0 to 4 months [16]. Breast milk from vegetarian mothers is nutritionally adequate and it has been associated with infants’ growth rate at the lower end of normal for the first six months of life [17], probably because vegetarian women tend to breastfeed for a longer period [18,19]. No data are available for infants breastfed by vegan mothers. In infants not breastfed following a vegan diet, protein malnutrition may occur [20], hence a rice protein-based infant formula supplemented with lysine, threonine, and tryptophan or a soy-based infant formula fortified with methionine is needed to ensure a normal growth [21]. Infants fed with soy-isolate drink, whether or not methionine supplemented, were thought to have similar growth rate compared to infants fed with cow’s milk formula [22]. Recently, non-fortified plant-based drinks (soy, cereal, etc.) have been shown to cause severe protein energy deficiencies and have been contraindicated in infancy [13].

• Vitamin B12

Infant’s vitamin B12 requirements range between 0.5 and 0.8 μg/day [16]. Vitamin B12 is almost exclusively present in products of animal origin, but it can also be found in some algae or fungi with limited bioavailability. Other foods, such as tempeh, can raise vitamin B12 levels, but it is not a sufficient source for vegans. Therefore, vitamin B12 supplementation is necessary for vegans at any age.

Long-term vegan mothers, with little or no supplementation, expose their exclusively breastfed infants to vitamin B12 deficiency [23] between 2 and 12 months of life [24]. Vitamin B12 concentration in breast milk and serum levels in breastfed infants are directly correlated [25], so that vitamin B12 supplementation with appropriate products in breastfeeding mothers is mandatory. Rice- or soy-based fortified infant formula have an adequate vitamin B12 content for infants. Vitamin B12 deficiency has been described in up to 45% of infants on a vegan diet [26] and, therefore, they must receive vitamin B12 since the beginning of weaning, unless they consume a rice- or soy-based infant formula, as previously stated.

Vitamin B12 deficiency in infants may be responsible of anemia and developmental delay, anorexia, failure to thrive, neurological symptoms (e.g., involuntary movements, abnormal EEG), palmar/plantar hyperpigmentation [27,28]. In children greater than six months, the high consumption of folates with vegetarian and vegan diets can hide the hematological features of vitamin B12 deficiency and neurological symptoms are usually the first signs. 

• Calcium and Vitamin D

The calcium needs of infants below the age of 12 months are 500 mg/day, whereas the vitamin D daily requirement is 10 μg/day (or 400 IU/day) [16]. However, vitamin D requirement depends also on the exposure to sunlight, since vitamin D deficiency is more likely in dark-skinned people and at high latitudes. Adequate calcium and vitamin D intakes are critical in infancy to ensure bone growth and mineralization. As reported by Dagnelie et al. [29], calcium and vitamin D levels were significantly decreased in macrobiotic infants at 10-20 months compared to omnivores, with a higher incidence of rickets (55% vs. 0%). Low bone mineral density and a greater risk of fractures have been described since young age in adolescents on macrobiotic or vegan diet low in calcium [30]. Bone mineral density and risk for bone fractures do not differ in omnivores and lacto-vegetarians [31,32]. 

Calcium is mainly provided by dairy products; however, some plants, soy drinks, and tofu are other possible sources, but calcium bioavailability is inversely related to phytate and oxalate content [33,34]. Green vegetables poor in oxalate (e.g., broccoli, kale and cabbage) are good sources of calcium, whereas nuts, dried beans and spinach contain calcium with low bioavailability [17]. Vitamin D derives almost exclusively from fatty fish and fortified products and vegan children, if not adequately supplemented, are particularly at risk for vitamin D deficiency. 

Infants breastfed by vegan mothers receive enough calcium, because maternal calcium absorption is enhanced in response to higher 1,25-dihydroxy vitamin D blood concentrations [35] and calcium is mobilized from the mother’s bones [8]. Non-breastfed infants fed with plant-based drinks may be exposed to calcium deficiency with seizures [13,24]. Calcium-supplemented rice- or soy-based infant formulas are a safe alternative. 

A nutritional source of vitamin D, i.e., fortified soy drinks, dairy products or cereals, is necessary for people not routinely exposed to sunlight, users of sunblock and dark-skinned individuals [17]. To prevent vitamin D deficiency, the AAP recommends that all breastfed infants are given 400 IU/day of vitamin D from the first few weeks of life. The only vitamin D drops for infants are in the form of vitamin D_3_, from lanolin of sheep’s wool, not accepted by vegans. In case of refusal by vegan parents, an alternative under investigation is the supplementation of the breastfeeding mother with vitamin D_2_ (derived from fungi) at 2000 IU/day or 60,000 IU/month for three months, along with proper sunlight exposure [11]. 

• Iron

The recommended iron requirements in the first year of life range from 6 to 8 mg/day [16]. Vegetarian and vegan diets contain good amounts of iron but its bioavailability from plant is lower compared to animal sources (2–5% vs. 20–30%) [36]. However, it is enhanced by many components of both fruit and vegetables: carotenes, retinol, and acids such as ascorbic, citric, lactic, malic, and tartaric. Soaking pulses and flour activate phytases increasing iron absorption. 

During pregnancy and breastfeeding, maternal iron deficiency is common and the majority of women require iron supplementation after careful monitoring of their status [37]. No significant differences have been found in breast milk content of iron between vegetarian/vegan and omnivore mothers [38]. It is well known that breast milk is not a good source of iron during the first 4–6 months [36], as demonstrated by the relatively high incidence of mild anemia in infants, but there is no supporting evidence for daily iron supplementation during exclusive breastfeeding [39].

Infants of vegetarian/vegan mothers should have their iron status monitored and their mothers should be encouraged to consume iron-rich foods [17,40]. Iron daily intake is not a concern in formula-fed infants of vegetarian/vegan mothers, because infant formulas are supplemented with iron. Breastfed infants of vegetarian/vegan mothers should be weaned with iron-fortified foods (e.g., cereals) [11].

The incidence of iron deficiency anemia during weaning is not increased in vegetarian/vegan infants and their serum ferritin levels, as well as growth, are not reduced compared to omnivores [41,42]. However, a previous study found that vegetarian children under three years of age had serum ferritin levels < 10 mg/L in 64% of cases, hence these findings should be interpreted with caution [43].

• Zinc

The recommended zinc requirements in the first year of life are 5 mg/day [16]. More than 50% of the zinc intake derives from animal products, either meat or dairy products [44,45], and some plants (e.g., cereals, whole seeds, legumes, nuts) [46], but phytates and oxalates reduce its absorption [47,48]. Some substances in fruits, such as organic acids, sulfur-containing amino acids, peptides containing cysteine or hydroxy acids [49] and different procedures, like fermentation, soaking, grinding, or sour-dough leavening, increase zinc absorption [50,51]. Zinc absorption from vegetarian/vegan diets is around 15–26%, and 33–35% from omnivore diets [47,52].

Zinc deficiency in infants may cause failure to thrive, taste changes, and increased susceptibility to infection; if severe, diarrhea and mucocutaneous damage, including dermatitis and alopecia, are reported.

Zinc levels in breast milk are not influenced by maternal diet and zinc status of breastfed infants do not differ if the mothers are vegetarian/vegan or omnivore [40]. Zinc-fortified cereals are a good source of zinc during weaning, mainly for breastfed infants [11]. Recently, a review showed that zinc levels are similar between vegetarians and omnivores during weaning, but growth, cognitive development, and the occurrence of infections were not evaluated [53]. 

• Iodine

The recommended iodine daily intake in the first 12 months of life ranges between 50 and 80 μg/day [16]. Seafood and dairy products contain large amount of iodine; however, iodized salt remains the best way to meet iodine needs in breastfeeding women, especially in vegan mothers whose mean iodine daily intake is around 30 μg/day, below the recommended threshold [54]. During pregnancy and lactation, 5 to 6.5 g of iodized salt per day provide about 200 μg per day of iodine [9]. In infants, iodized salt is not advised before 12 months of age [10] and 400 ml of breast milk or 900 ml of infant formula supply a sufficient amount of iodine [55,56,57]. After 12 months, the recommended intake ranges between 2 and 5 g/day depending on the iodine content of salt, which differs in each country [9,54].

• Long-chain Polyunsaturated Fatty Acids (LC-PUFA)

Docosahexaenoic acid (DHA, 22:6 n-3) and eicosapentaenoic acid (EPA, 20:5 n-3) have a significant role in pre- and postnatal brain development, retinal function, behavior and mood [58].

DHA and EPA are mainly present in seafood products, whereas vegetable sources are limited to some algae [59,60,61]. Among n-3 fatty acids, α-linolenic acid (ALA, 18:3 n-3) is the only one in good amounts in plants, seeds (e.g., hemp and chia seeds), walnuts, and certain algae [61]. 

ALA is an essential fatty acid, whereas DHA and EPA can be synthesized from ALA. Nonetheless, the synthesis of DHA and EPA from ALA is hindered and influenced by several factors, such as dietary intake of linoleic acid [62], energy, protein, pyridoxine, biotin, calcium, copper, magnesium, zinc [63,64], trans fatty acids [61] and alcohol [65].

A very high omega-6 or -9 (oleic acid) consumption could alter omega-6/-3 and omega-9/-3 ratios, reducing ALA conversion to DHA and EPA. Accordingly, the European Food Safety Authority recommends a minimum intake of DHA and/or EPA from 0 to 18 years of age [66]. Since vegan diets provide insufficient amounts of LC-PUFA, oils rich in ALA should be preferred (walnuts, rapeseed, soy), whereas those with a high linoleic acid/ALA ratio (peanut, corn, sunflower) or rich in omega-9 (olive) should be limited. 

Vegetarian pregnant women have lower blood levels of DHA compared to omnivore ones, but offspring’s growth data are reported to be in the normal range [67]. Lower DHA levels have been found in breast milk from vegetarian/vegan mothers in comparison to omnivore [68], suggesting the need to supplement with 100–200 mg/day of DHA during pregnancy and lactation. DHA from algae represent an acceptable option for vegan mothers.

Infants from 6 to 12 months on vegetarian/vegan diet should continue to consume breast milk or infant formula on demand with oils rich in omega-3 (rapeseed, walnut, soybean) added on one or two meals/day as sources of DHA.

• Fiber

Plant foods are rich in fiber that are components not digestible by human enzymes in the gut. Fiber may be either fermented by gut bacteria or increase the volume of ingested food, thus reducing the intake of protein, fat, and calories. Therefore, an excessive consumption of fiber during late pregnancy, lactation and infancy may be detrimental due to the reduction of food, nutrients and energy intake [9,54]. Vegan meals during pregnancy, lactation, and infancy up to 12 months of life should be low on fiber and rich in fruits and vegetable juices, peeled beans, refined grains, and soy derivatives (e.g., milk, tofu, and yoghurt).

• Practical Recommendations

Practical recommendations regarding breastfeeding, formula feeding and complementary foods in either vegetarian or vegan mother–baby dyads based on current evidence have been proposed by Lemale et al. [8] and Mangels et al. [11] (Table 5).

## 4. Discussion

The prevalence of alternative feeding regimens in either adults or infants in our study sample is 8.6%, similar to what has been reported [1]. Mothers following a vegetarian/vegan diet are more numerous than fathers (8.6% vs. 2.8%) and closer to the percentage of infants weaned according to alternative feeding regimens (9.2%). We believe that maternal feeding habits have a greater influence on infant weaning, as confirmed by the finding that in the group of infants following an alternative weaning regimen there are 51.5% vegetarian/vegan mothers compared with 27.3% fathers.

Breastfeeding lasts longer in the alternative weaning group (15.8 vs. 9.7 months; *p* < 0.0001), probably because vegetarian/vegan mothers perceive breast milk as more natural and safer for their offspring.

Half of the families do not perceive their pediatrician as an appropriate guide in leading alternative weaning regimens and this lack of surveillance, together with the unsatisfactory nutritional knowledge in alternative feeding regimens showed by a significant percentage of pediatricians [69], may expose infants to a serious risk of severe nutritional deficiencies.

Since a vegetarian diet has a smaller variety of foods than an omnivore one and a vegan diet is even more restrictive, neonates whose mothers are vegetarian/vegan and infants following alternative weaning methods are likely to suffer from clinical or sub-clinical nutritional deficiencies.

To date, consistent findings to support both safety and feasibility of alternative weaning methods are still lacking. Data in infancy and childhood are scarce, since only few case reports and studies in adults are currently available. Importantly, no randomized controlled trials or observational studies were found, drastically reducing robustness of evidence and signaling the need for future research.

Many scientific societies [8,10] promote diet regimens made of large variety of foods and do not support alternative weaning methods because of nutritional deficiencies and their effects. Alternative weaning regimens should not be an option for ex-preterm infants, because of the absence of any guidelines in this high-risk population [5,70].

Despite this position, some authors claim that a vegan diet would be appropriate in every phase of life, from pregnancy to adulthood [9] and a growing number of families follow vegetarian or vegan feeding regimens. 

Alternative weaning as a self-decision should be generally discouraged. Pediatricians should guide families strongly willing to follow a vegetarian/vegan regimen, providing all nutritional requirements. A close follow-up of the infant is mandatory, keeping in mind that the more restricted the diet, the higher the risk of possible deficiencies. During very delicate stages of life, such as pregnancy, lactation and infancy, cooperation and correct information by an expert pediatrician or nutritionist are needed to provide the highest degree of nutritional care through counselling, monitoring possible nutritional deficiencies, and prescribing supplements. 

A well-planned alternative diet [9] during late pregnancy, lactation, and infancy should meet the following criteria:

- Consume large amounts and a wide variety of plant foods, better whole or minimally processed foods;
- Limit the amount of fiber;
- Choose vegetable fats cautiously, favoring sources of omega-3 fatty acids and monounsaturated oils, and limiting trans-saturated fats and tropical oils (e.g., coconut and palm) to preserve omega-3 metabolic pathway; during infancy and early childhood fats intake should not be restricted;
- Consume adequate amounts of calcium from calcium-rich foods (dairy products for lacto-ovo-vegetarians and lacto-vegetarians; calcium-rich plants, soy drinks, and tofu for ovo-vegetarians and vegans);
- Supplement vitamin D (1000–1200 IU/day if breastfed, or 600-800 IU/day if formula fed with vitamin D-enriched formula);
- Consume adequate amounts of vitamin B12 (from fortified rice- or soy-based infant formula -if not breastfed-, algae, some fungi, tempeh) or supplement the lactating mother and the infant.

During vegetarian or vegan weaning, breast milk is the main caloric source for the neonate, hence maternal diet should include vitamin B12 supplementation and the right intake of vitamin D, calcium and essential fatty acids. If the infant is formula-fed, rice- or soy-based infant formula provide right amounts of vitamin B12, calcium, iron, zinc, iodine and DHA [8].

A vegetarian or vegan weaning should replace meat with high protein-rich foods, such as pulses, legumes, soy and soy derivatives (milk, yoghurt, tofu). Dried fruits or smashed oily seeds added to baby food and yoghurt (or soy yoghurt if vegans) increase the variety of proteins with correct caloric intake. At the start of weaning, baby foods are creamy, easy to chew, with iron- and zinc-enriched cereals and no fiber in order to increase absorption. As far as flours are concerned, those enriched with iron and calcium (iron, 10 mg/100g; calcium, 400–560 mg/100g) should be preferred to reduce the risk of anemia and altered bone metabolism. The addition of few drops of lemon, as a source of vitamin C, increases the absorption of iron.

Another issue is the low availability of commercial baby foods for vegetarian or vegan weaning; plain fruits and vegetables puree, plain cereals and few combination dishes are produced. Moreover, some vegetarian/vegan commercial foods contain animal-derived gelatin, meat and fish oil. As a consequence, vegetarian parents prefer to prepare their own infant foods, but this could expose the infant to not well-balanced preparations, i.e., high in sodium, but poor in energy, protein, fat, iron, and zinc [71]. Soy products, dried beans, wheat germ, avocado, ground nuts, and nut butters are all good sources of these nutrients and their consumption should be emphasized. 

## 5. Conclusions

Weaning is a critical moment and should be implemented under pediatric supervision that becomes critical in case of vegetarian/vegan parents. To date, consistent findings to support both safety and feasibility of alternative weaning methods are still lacking, since only few case reports and studies conducted in adults are currently available.

Vegetarian weaning with appropriate guidance from family pediatricians or nutritional experts is possible and it should not be opposed.

Vegan weaning should be discouraged because serious damages (slow growth, rickets, irreversible cognitive deficits, cerebral atrophy, and also death) have been demonstrated.

Alternative weaning regimens should be contraindicated for ex-preterm infants. The pivotal role of pediatricians to guide parents on the more appropriate diet regimen during infancy and childhood requires a proper training on nutrition.

Communication between parents and pediatricians is essential: health professionals need to spend time to explain any possible consequences of a diet lacking vitamin D, vitamin B12, zinc, iron, folates, omega-3, LC-PUFA, proteins and calcium. Pediatricians need to be aware of even subtle signs and symptoms due to nutritional and metabolic deficiencies to start treatment as soon as possible. 

Intermittent infant blood testing may be useful to determine micronutrient deficiency but may not be acceptable to some families. Referral to a nutritionist acquainted with alternative feeding regimens diets can be indicated, since a shared partnership between the family pediatrician, the nutritionist and parents may be extremely helpful to carefully review dietary intake, including all supplements, and to ensure appropriateness of the chosen feeding regimen.

## Figures and Tables

**Table 1:** Demographic features.

Demographic Features		TOTAL 360
**Maternal Education**	Primary school Middle school High school Higher education	1 (0.3%) 23 (6.4%) 133 (36.9%) 203 (56.4%)
**Paternal Education**	Primary school Middle school High school Higher education	1 (0.3%) 46 (12.8%) 177 (49.2%) 136 (37.7%)
**Infant Gender**	Male Female	194 (53.9%) 166 (46.1%)
**Pregnancy**	Physiologic Complicated	323 (89.7%) 37 (10.3%)
**Neonate**	Healthy Inborn diseases (malformations or hereditary issues)	347 (96.4%) 13 (3.6%)

**Table 2:** Main outcomes.

**Maternal Diet Regimen**	Omnivore Alternative - Semi-vegetarian - Lacto-ovo-vegetarianism - Vegan - Fruit diet	329 (91.4%) 31 (8.6%) - 9 (2.5%) - 13 (3.6%) - 8 (2.2%) - 1 (0.3%)
**Paternal Diet Regimen**	Omnivore Alternative - Semi-vegetarian - Lacto-ovo-vegetarianism - Vegan - Fruit diet	350 (97.2%) 10 (2.8%) - 1 (0.3%) - 4 (1.1%) - 5 (1.4%) - 0 (0%)
**Infant Weaning**	Omnivore Alternative - Semi-vegetarian - Lacto-ovo-vegetarianism - Lacto-vegetarianism - Vegan	327 (90.8%) 33 (9.2%) - 16 (4.5%) - 7 (1.9%) - 2 (0.6%) - 8 (2.2%)
**Weaning Time (Months)**	<4 4–6 >6	10 (2.8%) 175 (48.6%) 175 (48.6%)
**Milk Type**	Breast milk Infant Formula Mixed (breast + formula) Cow’s milk	173 (48.1%) 48 (13.3%) 133 (36.9%) 6 (1.7%)
**Breastfeeding Duration (Months, Mean)**	Omnivores Alternative	9.7 15.8 *p* < 0.0001
**Child Diet after Weaning**	Omnivore Alternative - Semi-vegetarian - Lacto-ovo-vegetarianism - Vegan	341 (94.7%) 19 (5.3%) - 7 (2.0%) - 4 (1.1%) - 8 (2.2%)

**Table 3:** Weaning and parental diet.

Weaning	Mother Diet	Father Diet
**Omnivore (*t* = 327)**	Omnivore 313 (95.7%) Alternative 14 (4.3%)	Omnivore 326 (99.7%) Alternative 1 (0.3%)
**Alternative (*t* = 33)**	Omnivore 16 (48.5%) Alternative 17 (51.5%)	Omnivore 24 (72.7%) Alternative 9 (27.3%)

**Table 4:** Studies included in the narrative review.

Authors	Article Type	Year	Age of the Population	Main Conclusions
Lemoine et al. [12]	Case report	2020	13 months	Warning against nutritional deficiencies due to vegetarian/vegan diets in infants and children.
Lemale et al. [8]	Recommendations GFHGNP	2019	Infancy, childhood and adolescence	Vegan diets are not suited to children; strict guidance of competent health professionals is needed to prescribe nutritional supplements essential to their dietary balance.
Lemale et al. [13]	Brief report	2018	0–1 year	Replacing infant formula with nondairy drinks may lead to severe adverse effects, especially if early in life.
Baroni et al. [9]	Recommendations SSNV	2018	Pregnancy, lactation, infancy and childhood	Vegetarian/vegan diets are suitable during pregnancy, lactation, infancy, and childhood, but special attention to critical elements (e.g., protein, vitamin D and B12, fiber, omega-3 fatty acids, iron, zinc, iodine, calcium) is needed.
Fewtrell et al. [10]	Position paper ESPGHAN	2017	n.a.	Vegan diets are generally contraindicated during weaning due to the high risk of severe deficiencies.
Ferrara et al. [1]	Editorial	2017	0–2 years	Vegetarian or vegan diets need appropriate pediatric supervision to guarantee adequate supply of nutrients.
Agnoli et al. [4]	Position paper SINU	2017	Pregnancy, lactation, pre-scholar age, children, adolescents, adults, and the elderly	Well-planned vegetarian diets may provide adequate nutrient intake. Special attention should be made during pregnancy, breastfeeding and infancy, since nutritional deficiencies have been widely reported.
Mangels et al. [11]	Recommendations	2012	Infancy and early childhood	Carefully planned vegetarian and vegan diets are adequate for infants and toddlers.
Van Winckel et al. [14]	Review	2011	Infants, toddlers/preschool children, adolescents	Lacto-ovo-vegetarian diets may be appropriate for the growing child, whilst a vegan diet requires supplementation with vitamin B12. Special care is needed for the intakes of calcium, zinc and high-quality protein. The risk for deficiencies is inversely related to the age of the child and the variety of the foods.

**Table 5:** Practical recommendations for infant feeding during the first year of life according to vegetarian or vegan regimens (sources: Lemale et al. [8] and Mangels et al. [11]).

	Vegetarian Diet			Vegan Diet	
BF (Lactating Woman)	FF	CF	BF (Lactating Woman)	FF	CF
**Vitamin B12:** is likely to be lacking. Vitamin B12 fortified foods (cereals, alternative milk, meat analogs, and nutritional yeast) or vitamin B12 supplementation (50 μg/day) are recommended. If the mother refuses either, the baby should be supplemented with vitamin B12. **Vitamin D:** vitamin D supplementation of 1000–1200 IU/day is recommended in all breastfed infants. Currently, the only commercial infant vitamin D drop is derived from sheep’s wool (lanolin), thus suitable for vegetarians but not for vegans. **Proteins:** Varied consumption of vegetables and cereals. **Calcium:** 500–1000 mg/day depending on other sources. **Iron:** Iron-rich plants with vitamin C-rich fruit. Specific preparation methods (grinding, soaking, germination). Supplementation of 2–3 mg/kg of iron depending on serum ferritin. **Zinc:** Plants rich in zinc (Brassicaceae); Specific preparation methods (grinding, soaking, germination). If deficiency: 1 mg/kg/day of zinc gluconate. **Iodine:** 6.5 g/day of iodized salt. **DHA and ALA:** Vegetarian lactating women should consume DHA fortified foods and ALA rich foods (flaxseeds, chia seeds, walnuts, etc.). 100–200 mg of micro-algae/day source of DHA suitable also for vegans.	Cow’s milk-based formula provides good amounts of Vitamin B12, proteins, calcium, iron, zinc, iodine, and DHA (if DHA enriched). **Vitamin D:** 1000–1200 IU/day (or 600–800 IU/day if formula is vitamin D enriched). **DHA:** if formula not DHA enriched, 100 mg of micro-algae/day	In formula-fed infants, there are no specific concerns. Conversely, breastfed infants may lack iron and zinc, therefore iron- and zinc-fortified infant cereal or firm tofu should be advised. **Iodine:** No addition of iodized salt up to 12 months of life.	**Vitamin B12:** same considerations as for vegetarian diet. **Vitamin D:** if the family declines to supplement the baby with lanolin-derived vitamin D3, the mother should receive a high-dose vitamin D2 (derived from fungi) supplementation of 2000 IU/day or 60,000 IU/month for three months to ensure good concentrations of vitamin D in breast milk. **DHA and ALA:** same considerations as for vegetarian diet.	Soy protein-based formulas (even though they are supplemented with lanolin-derived vitamin D3). Commercial soy drinks or other plant-based beverages, homemade formulas from grains or nuts, vegetable juice, and unmodified cow’s milk should be avoided. **Vitamin D:** 1000–1200 IU/day (or 600–800 IU/day if formula is vitamin D enriched) **DHA:** if formula not DHA-enriched, 100 mg of micro-algae/day	Same considerations as for vegetarian diet.

BF: breastfeeding; FF: formula feeding; CF: complementary food; DHA: Docosahexaenoic acid; ALA: α-linolenic acid.
